# Construction and characterization of the Korean whole saliva proteome to determine ethnic differences in human saliva proteome

**DOI:** 10.1371/journal.pone.0181765

**Published:** 2017-07-24

**Authors:** Ha Ra Cho, Han Sol Kim, Jun Seo Park, Seung Cheol Park, Kwang Pyo Kim, Troy D. Wood, Yong Seok Choi

**Affiliations:** 1 College of Pharmacy, Dankook University, Cheonan, Chungnam, South Korea; 2 Department of Applied Chemistry, The Institute of Natural Science, College of Applied Science, Kyung Hee University, Yongin, Kyoungki, South Korea; 3 Department of Chemistry, The State University of New York at Buffalo, Buffalo, New York, United States of America; CHA University, REPUBLIC OF KOREA

## Abstract

As the first step to discover protein disease biomarkers from saliva, global analyses of the saliva proteome have been carried out since the early 2000s, and more than 3,000 proteins have been identified in human saliva. Recently, ethnic differences in the human plasma proteome have been reported, but such corresponding studies on human saliva in this aspect have not been previously reported. Thus, here, in order to determine ethnic differences in the human saliva proteome, a Korean whole saliva (WS) proteome catalogue indexing 480 proteins was built and characterized through nLC-Q-IMS-TOF analyses of WS samples collected from eleven healthy South Korean male adult volunteers for the first time. Identification of 226 distinct Korean WS proteins, not observed in the integrated human saliva protein dataset, and significant gene ontology distribution differences in the Korean WS proteome compared to the integrated human saliva proteome strongly support ethnic differences in the human saliva proteome. Additionally, the potential value of ethnicity-specific human saliva proteins as biomarkers for diseases highly prevalent in that ethnic group was confirmed by finding 35 distinct Korean WS proteins likely to be associated with the top 10 deadliest diseases in South Korea. Finally, the present Korean WS protein list can serve as the first level reference for future proteomic studies including disease biomarker studies on Korean saliva.

## Introduction

Saliva is secreted from salivary glands, including three major glands (parotid, submandibular, sublingual glands) and minor glands. Saliva has various functions. It maintains oral cavity homeostasis, lubricates oral tissues, promotes chewing, swallowing, digestion, and speaking, and protects the oral cavity against microorganisms [1–4]. It is composed of water, proteins, peptides, lipids, other small molecules, and minerals. Healthy adults are known to produce 500–1500 mL of saliva daily at a rate of about 0.5 mL/min [1, 4]. Most organic compounds in saliva are produced in the salivary glands, but some are transferred from blood through various mechanisms, including diffusion, active transport, and ultrafiltration [4]. Moreover, its collection is non-invasive and it is easy to collect and store saliva samples [5]. Thus, saliva can be a good alternative to blood for diagnosis due to its characteristics mentioned above. For example, major systemic infections of viruses such as human immunodeficiency virus, hepatitis C virus, and human papillomavirus have been successfully tested by saliva-based diagnostic methods [6]. Thus, clinical diagnosis using saliva specimens is an emerging field. Among various constituents of saliva, proteins have gained the most interest as probable disease biomarkers because numerous proteins are known to be present in the saliva and many of them are believed to represent the progress of diseases [4].

As the first step to discover protein disease biomarkers from saliva, global analyses of the saliva proteome have been carried out since the early 2000s. As a result, more than 3000 proteins have been identified in human saliva [1, 7–11]. Some of them are accessible through public databases such as Human Salivary Proteome Central Repository (1,166 proteins) and Sys-BodyFluid Database (2,161 proteins) [12, 13]. Additionally, systematic comparisons of human saliva and plasma proteomes have been carried out and several interesting points have been reported in the saliva proteome [1, 2]. First, only about 27% of proteins identified in human whole saliva (WS) are found in plasma, indicating that it is possible to discover totally novel biomarkers from saliva [2]. In addition, human saliva and plasma proteomes are over-represented in the categories of response-to-stimulus and response-to-stress compared to total human proteome. This indicates that both fluids (saliva and plasma) might play important roles of in the defense system of the human body and their probable potential for disease diagnosis [1, 2]. These points have been supported by the discovery of many protein disease biomarker candidates from saliva for oral diseases and systemic diseases [2, 5, 14–17]. Moreover, about 58% of immunoglobulins (Igs) identified in human saliva are found in plasma, and the abundance of these overlapping Igs in saliva and plasma shows a high correlation (r of at least 0.87) [1, 2]. This indicates that it is possible to transform antibody-based diagnostic methods using blood to methods employing saliva; an excellent example is the commercial saliva HIV test kit [6].

Recently, ethnic differences in human plasma proteome have been reported. Jeong et al. confirmed 100 unique proteins out of 185 proteins in Korean plasma compared to 3,380 proteins in the HUPO Plasma Proteome Project dataset [18], and Kim et al. observed plasma level differences of some cardiovascular disease protein marker candidates between African-American and non-Hispanic White ethnicity [19]. These results indicate that there is a fundamental need to determine ethnic difference in human saliva proteome. Unique proteins might be found only in ethnicity-specific saliva samples and they might be useful as novel biomarkers for diseases prevalent in that ethnic group.

Therefore, the Korean WS proteome catalogue indexing 480 proteins was built and characterized in this study through proteomic analyses of WS samples collected from eleven healthy Korean male adult volunteers for the first time. It was then compared to the integrated human saliva proteome including 3,449 proteins to determine ethnic differences in human saliva proteome. Confirmed differences of protein identities and GO category distributions between the two proteomes strongly support that there are ethnic differences in the human saliva proteome. In addition, some distinct proteins in the Korean WS are likely to be associated with highly prevalent diseases in South Korea, demonstrating the high diagnostic potential of ethnicity-specific human saliva proteins for diseases highly prevalent within an ethnic group. Finally, the present list of Korean WS proteins can serve as the first level reference for future proteomic studies including disease biomarker studies on Korean saliva.

## Materials and methods

### Sample collection and Preparation

This study was approved by Dankook University Institutional Review Board. The participants of this study were recruited between August 1, 2014 and Augst 31, 2014 by posting its notices including the brief summary of this study around Dankook University, Cheonan, Chungnam, South Korea. A total of 15 volunteers wanted to participate this study, but 4 of them were excluded based on their history of diseases informed through their introductory screening surveys. Finally, eleven healthy South Korean male adults (25.9±2.3 years old; 22–30 years old) were decided to be the participants and each participant signed an informed consent form. Any specific baseline demographic characteristics of the study populations were not available in this study, because only basic personal information and history of diseases were obtained through the introductory screening survey. WS (15 mL/person) was collected from volunteers at 9:30 am prior to eating and after rinsing the mouth with water. A protease inhibitor cocktail solution (Sigma-Aldrich, St. Louis, MO) was spiked (the final volume ratio of 1:100) to WS samples immediately after sample collection. These protease-spiked samples were centrifuged at 12,000 rpm and 4°C for 10 min. Each supernatant was stored at -70°C until use. Prior to protein digestion, 4 mL of the thawed protease-spiked sample supernatant was applied to a 3 kDa cutoff filter unit (Amicon Ultra-4, Merck Millipore, Billerica, MA) for buffer exchange with water. The filter unit was centrifuged at 3,500 rpm and the retentate was dried by vacuum centrifugation. The dried residue was resuspended with 500 μL of water and its total protein concentration was determined by BCA assay (Pierce BCA Protein Assay Kit, Thermo Scientific, Waltham, MA). An appropriate portion of the resuspended solution (equivalent to 1 mg of total protein) was then dried by vacuum centrifugation again, and the resulting residue was applied to procedures described previously with slight modifications (S1 Appendix) [20, 21]. A portion of the final form of the sample solution was subjected to nanoliquid chromatography-quadrupole-ion mobility spectroscopy-time of flight (nLC-Q-IMS-TOF) analysis. In the case of the pooled Korean WS sample, 1 mL of each thawed protease-spiked sample supernatant was mixed and the mixture was applied to the same method mentioned above. A portion of the final form of the pooled sample solution was subjected to nLC-Q-IMS-TOF analysis and nLC-Q-orbitrap analysis.

### Separation and analysis

All nLC-Q-IMS-TOF analyses were carried out on a Waters nanoACQUITY UPLC system (Waters, Milford, MA) and a Waters SYNAPT G2-S HDMS system. The prepared sample was injected into a Waters nanoACQUITY UPLC Symmetry C18 trap column (5 μm, 0.18×20 mm). It was desalted with 99% mobile phase A (0.1% formic acid in water) and 1% mobile phase B (0.1% formic acid in acetonitrile) for 5 min at a flow rate of 10 μL/min. Trap column-retained peptides were eluted into a Waters nanoACQUITY UPLC BEH300 C18 column (1.7 μm, 0.075×250 mm) and separated by a linear gradient of mobile phase B from 1 to 60% for 120 min at a flow rate of 250 nL/min. Peptides eluted from the analytical column were delivered into the mass spectrometer through a nanoelectrospray ionization (nESI) source operating in positive ion mode. Mass spectrometry of peptide ions was performed in resolution data-independent acquisition mode (MS^E^). Prior to fragmentation processes, IMS was carried out to separate similar precursor ions. Parameters related with mass spectrometry are listed in S1 Appendix.

All nLC-Q-orbitrap analyses were carried out on a Thermo Scientific Easy-nLC 1000 system (Waltham, MA) and a Thermo Scientific Q Exactive system. The prepared sample was desalted by Top Tip (Glygen, Columbia, MD) following the direction by the manufacturer and the desalted sample was separated on an in-house analytical column (0.075×250 mm), packed with C18 resin (Jupiter, 3 μm, Phenomenex, Torrance, CA), by a linear gradient of mobile phase B from 1 to 80% for 120 min at a flow rate of 300 nL/min. Peptides eluted from the column were delivered into the mass spectrometer through a nESI source operating in positive ion mode. Mass spectrometry of peptide ions was performed in data-dependent product ion scan (MS^2^). Parameters related with mass spectrometry are listed in S1 Appendix.

### Protein identification and bioinformatics

Raw data from nLC-Q-IMS-TOF and nLC-Q-orbitrap were analyzed with Waters ProteinLynx Global Server (PLGS) v3.0.2 and Thermo Proteome Discoverer v2.1, respectively. For the identification of peptides and proteins, database search against the IPI human database v3.87 was performed and database search parameters are listed in S1 Appendix. All database search results were verified manually.

For GO analysis of saliva proteomes, the Generic GO term mapper was used [22]. Significance of difference in individual GO categories between the Korean WS proteome and the integrated human saliva proteome was tested by the chi-square method [23].

Database of disease-related biomarkers was used to check probable association between distinct proteins observed in Korean WS proteome but not in the integrated human saliva proteome and diseases [24].

## Results

### The Korean WS proteome

**Fig 1:**
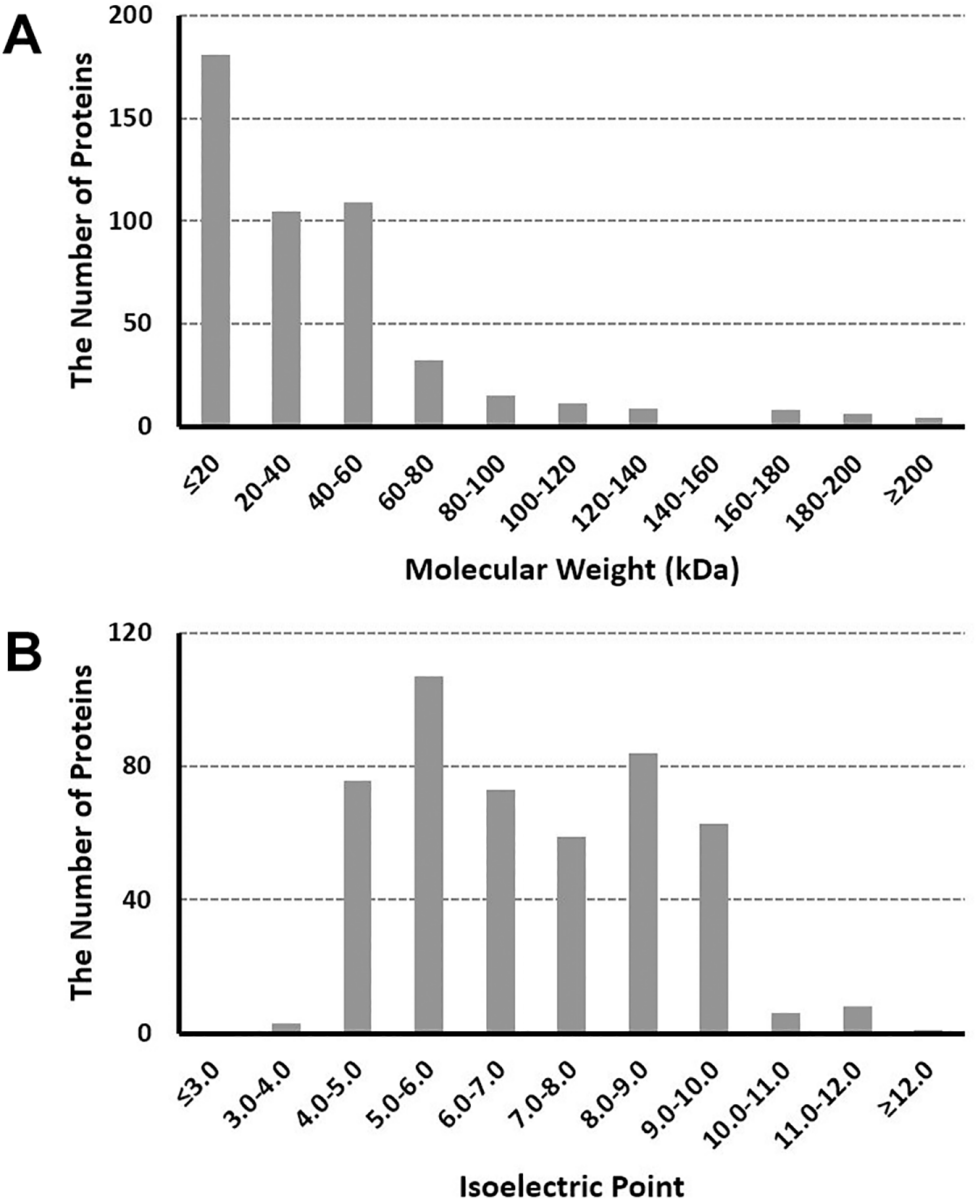
Theoretical molecular weight (A) and isoelectric point (B) distribution of the Korean whole saliva proteome.

Based on nLC-Q-IMS-TOF analyses of Korean WS samples, the Korean WS proteome was built successfully for the first time. In order to enhance the credibility of protein identification results, the following criteria were set: 1) any identification derived from only one unique peptide was rejected, 2) FDR was kept at no more than 1%, 3) only protein identification with at least 95% probability from PLGS results were accepted, and 4) all results which passed the above criteria were verified manually. These criteria were applied to all downstream protein identifications. As a result, a total of 480 proteins were identified (S1 Table). Also, the distribution of theoretical molecular weight and isoelectric point (pI) of the Korean WS proteome were examined (Fig 1A for molecular weight and Fig 1B for pI). As shown in Fig 1A, a large portion (82.3%) of the Korean WS proteome is composed of proteins with molecular weight of less than 60 kDa. There is a roughly inverse correlation between distribution proportions and molecular weights of component proteins at range of 60–160 kDa. In the case of pI distribution, the Korean WS proteome is composed of 16.5, 37.5, 30.0, and 16.0% of proteins with pI values lower than 5.0, between 5.0 and 7.0, between 7.0 and 9.0, and higher than 9.0, respectively (Fig 1B). The average molecular weight and pI value of the Korean WS proteome were calculated to 42 kDa and 6.95, respectively.

### Comparison of protein lists from the Korean WS proteome and the integrated human saliva proteome

**Fig 2:**
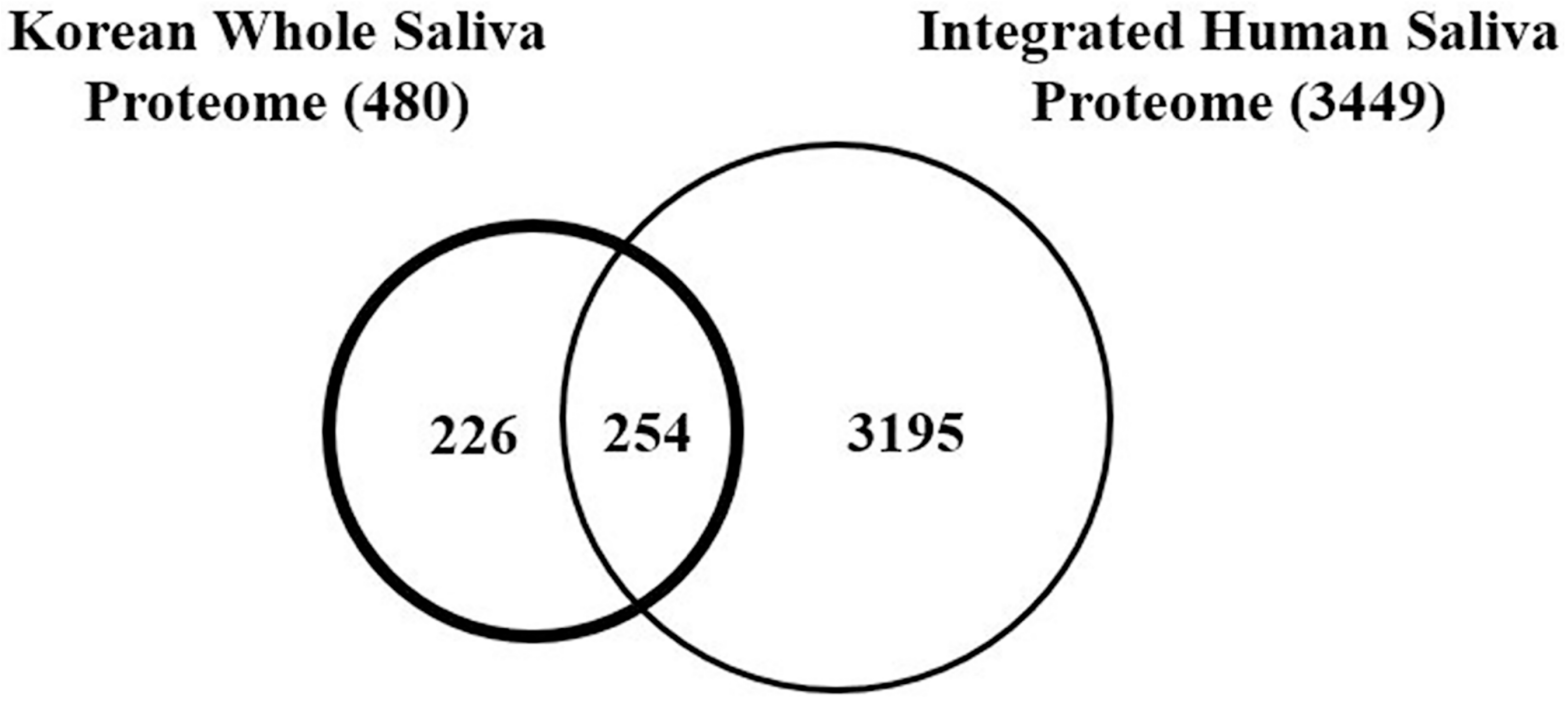
Venn diagram illustrating the total number of proteins specific to either the Korean whole saliva proteome or the integrated human saliva proteome and those observed in both proteomes.

To determine ethnic differences in the human saliva proteome, the present Korean WS proteome was compared to P. Sivadasan et al.'s updated human saliva protein list (a total of 3,449 proteins) built by the integration of their own and previously-reported five human saliva protein lists [1, 7–11]. For accurate comparison between proteomes, all available information of a protein, including IPI accession number, SwissProt number, gene symbol, amino acid sequence, molecular weight, and brief description, was used for its UniProt KB search. Then, search results from similar proteins in various proteomes were carefully compared to one another to determine if they are the same. As shown in Fig 2, the Korean WS protein list has 226 out of 480 (47.1%) proteins not included in the integrated human saliva protein list. These distinct Korean WS proteins are summarized in Table 1 and S2 Table.

**Table 1:** Distinct proteins observed in Korean whole saliva but not in other human saliva. 1, response to stimulus; 2, response to stress; 3, cell communication; 4, protein metabolic; 5, other primary metabolic; 6, transport; 7, organization and biogenesis; 8, catabolic process; 9, cell homeostasis; 10, regulation of biological process; 11, nucleic acid binding; 12, protein binding; 13, other binding; 14, transporter activity; 15, signal transducer activity; 16, catalytic activity; 17, motor activity; 18, structural regulator; 19, transcription regulator; 20, antioxidant activity; 21, enzyme regulator activity.

Accession number	Description	Biological process	Molecular function	Biomarker candidates
IPI00000149	ISOFORM 1 OF CASPASE-8	1, 2, 3, 4, 6, 7, 8, 10	12, 13, 15, 16	o
IPI00003842	ISOFORM 1 OF MICROTUBULE-ASSOCIATED PROTEIN 2	7	12, 18	o
IPI00006079	ISOFORM 1 OF BCL-2-ASSOCIATED TRANSCRIPTION FACTOR 1	2, 3, 4, 5, 7, 10	11, 12	x
IPI00011528	CLEAVAGE STIMULATION FACTOR SUBUNIT 1	4, 5	11, 12	x
IPI00011651	ISOFORM 1 OF RECEPTOR-TYPE TYROSINE-PROTEIN PHOSPHATASE GAMMA	3, 4, 10	12, 15, 16, 18	x
IPI00012865	ISOFORM PLD1A OF PHOSPHOLIPASE D1	1, 3, 4, 5, 6, 7, 8, 10	12, 13, 16	x
IPI00015894	CDC42 EFFECTOR PROTEIN 4	3, 7, 10	12, 21	x
IPI00016472	ISOFORM 2 OF ZINC FINGER CCCH DOMAIN-CONTAINING PROTEIN 13	unknown	12, 13	x
IPI00017290	ISOFORM 5 OF SERINE/THREONINE-PROTEIN PHOSPHATASE 4 REGULATORY SUBUNIT 3A	unknown	unknown	x
IPI00018214	ISOFORM 1 OF PROTEIN MAX	1, 2, 3, 4, 5, 7, 10	11, 12, 19	o
IPI00019226	ISOFORM 1 OF BROMODOMAIN-CONTAINING PROTEIN 8	3, 4, 5, 7, 10	11, 12, 15, 18, 19	o
IPI00019794	SH3 AND MULTIPLE ANKYRIN REPEAT DOMAINS PROTEIN 3	1, 3, 4, 6, 7, 9, 10	12, 13, 18	x
IPI00020019	ADIPONECTIN	1, 2, 3, 4, 5, 6, 7, 8, 10	12, 13	o
IPI00022434	UNCHARACTERIZED PROTEIN (SIMILAR TO SERUM ALBUMIN)	unknown	unknown	o
IPI00024134	IG KAPPA CHAIN V-I REGION WALKER	3, 4, 6, 10	13, 16	x
IPI00026126	MAMMAGLOBIN-B	1, 3	12	o
IPI00027838	RNA-BINDING PROTEIN 4B	1, 5, 10	11, 12, 13	x
IPI00029403	SORTING NEXIN-4	1, 2, 3, 6, 7, 10	12, 13	x
IPI00030144	PEPTIDYL-PROLYL CIS-TRANS ISOMERASE A-LIKE 4A/B/C	4	16	x
IPI00032896	ISOFORM 1 OF DISRUPTED IN SCHIZOPHRENIA 1 PROTEIN	1, 3, 4, 7, 8, 9, 10	12, 18	x
IPI00033892	GDNF-INDUCIBLE ZINC FINGER PROTEIN 1	4, 5, 7, 10	11, 13, 19	x
IPI00064474	ZINC FINGER CCCH DOMAIN-CONTAINING PROTEIN 10	unknown	12, 13	x
IPI00102979	ORAL CANCER-OVEREXPRESSED PROTEIN 1	unknown	unknown	x
IPI00103636	ISOFORM 2 OF WAP FOUR-DISULFIDE CORE DOMAIN PROTEIN 2	4, 10	21	o
IPI00107502	ISOFORM 1 OF WD REPEAT AND SOCS BOX-CONTAINING PROTEIN 1	3, 10	12, 16	x
IPI00150554	UNCHARACTERIZED PROTEIN (SIMILAR TO CELL DIVISION CYCLE-ASSOCIATED 7-LIKE PROTEIN)	unknown	unknown	o
IPI00158280	FORMYLTETRAHYDROFOLATE DEHYDROGENASE ISOFORM A VARIANT	unknown	unknown	x
IPI00158847	DNAJ HOMOLOG SUBFAMILY C MEMBER 14	6	13	x
IPI00160396	ISOFORM 4 OF PUTATIVE HOMEODOMAIN TRANSCRIPTION FACTOR 2	4, 5, 10	11	x
IPI00169307	RHO GTPASE-ACTIVATING PROTEIN 21	3, 6, 7	12, 21	x
IPI00170867	ISOFORM 1 OF DEATH DOMAIN-ASSOCIATED PROTEIN 6	1, 2, 3, 4, 5, 7, 10	12, 13, 15, 21	x
IPI00181753	CDNA FLJ13286 FIS, CLONE OVARC1001154, HIGHLY SIMILAR TO HOMO SAPIENS CLONE 24720 EPITHELIN 1 AND 2 MRNA	unknown	unknown	o
IPI00185027	ISOFORM 1 OF ARGININE-GLUTAMIC ACID DIPEPTIDE REPEATS PROTEIN	4, 5, 6, 7, 10	11, 12, 13, 19	x
IPI00216773	UNCHARACTERIZED PROTEIN (SIMILAR TO SERUM ALBUMIN)	unknown	unknown	o
IPI00217345	ISOFORM 2 OF UDP-GLCNAC:BETAGAL BETA-1,3-N- ACETYLGLUCOSAMINYLTRANSFERASE 2	1, 4, 5, 7	16	x
IPI00217979	UNCHARACTERIZED PROTEIN MLL5	3, 4, 5, 7, 10	12, 13, 16	x
IPI00218573	ISOFORM 3 OF INTERLEUKIN-1 RECEPTOR ANTAGONIST PROTEIN	1, 2, 3, 5, 6, 7, 10	12, 13	o
IPI00218824	UBIQUITOUSLY TRANSCRIBED TETRATRICOPEPTIDE REPEAT PROTEIN Y-LINKED TRANSCRIPT VARIANT 29	4, 5, 7, 10	13, 16	x
IPI00220644	ISOFORM M1 OF PYRUVATE KINASE ISOZYMES M1/M2	1, 2, 4, 5, 7, 8, 10	11, 12, 13, 16	o
IPI00238220	UNCHARACTERIZED PROTEIN KIAA2022	7	unknown	x
IPI00246842	ISOFORM 1 OF TATA BOX-BINDING PROTEIN-ASSOCIATED FACTOR RNA POLYMERASE I SUBUNIT C	4, 5, 10	11, 12	x
IPI00291064	ISOFORM 1 OF AN1-TYPE ZINC FINGER PROTEIN 1	unknown	13	x
IPI00292753	ISOFORM 6 OF GTPASE-ACTIVATING PROTEIN AND VPS9 DOMAIN-CONTAINING PROTEIN 1	3, 6, 7	12, 21	x
IPI00299749	ZINC FINGER PROTEIN 16	4, 5, 7, 10	11, 12, 13, 19	x
IPI00300502	MYOZENIN-1	7	12	x
IPI00305457	PRO2275	unknown	unknown	o
IPI00328712	UNCHARACTERIZED PROTEIN C2ORF53	unknown	unknown	x
IPI00329002	SEC14 DOMAIN AND SPECTRIN REPEAT-CONTAINING PROTEIN 1	unknown	12, 13	x
IPI00329688	PROTEIN YIPF3	7	12	x
IPI00334190	STOMATIN-LIKE PROTEIN 2	2, 3, 4, 5, 6, 7, 9, 10	12, 13	x
IPI00376170	PEPTIDYL-PROLYL CIS-TRANS ISOMERASE A-LIKE	4, 5, 6, 7, 10	12, 13, 16	o
IPI00382577	KAPPA 1 LIGHT CHAIN VARIABLE REGION	unknown	unknown	x
IPI00384361	ISOFORM 3 OF TRIGGERING RECEPTOR EXPRESSED ON MYELOID CELLS 2	1, 2, 3, 4, 6, 7, 10	12, 13, 18	x
IPI00384397	MYOSIN-REACTIVE IMMUNOGLOBULIN LIGHT CHAIN VARIABLE REGION	unknown	unknown	x
IPI00384404	RHEUMATOID FACTOR RF-ET9	unknown	unknown	x
IPI00385533	ISOFORM 4 OF IODOTYROSINE DEHALOGENASE 1	5	12, 16, 20	x
IPI00386134	IG LAMBDA CHAIN V-I REGION BL2	3, 4, 6, 10	13, 16	x
IPI00386136	IG LAMBDA CHAIN V-VI REGION WLT	3, 4, 6, 10	13, 16	x
IPI00411765	ISOFORM 2 OF 14-3-3 PROTEIN SIGMA	2, 3, 4, 6, 7, 10	12, 21	o
IPI00412407	SERPIN PEPTIDASE INHIBITOR, CLADE B (OVALBUMIN), MEMBER 4, ISOFORM CRA_A	unknown	unknown	o
IPI00412701	ZINC FINGER PROTEIN 517	4, 5, 10	11, 13	x
IPI00418164	ISOFORM 1 OF COILED-COIL DOMAIN-CONTAINING PROTEIN 30	unknown	unknown	x
IPI00418780	ISOFORM 3 OF REGULATOR OF G-PROTEIN SIGNALING 3	3, 4	12, 21	x
IPI00419253	ISOFORM 1 OF NCK-ASSOCIATED PROTEIN 5	unknown	unknown	x
IPI00423464	PUTATIVE UNCHARACTERIZED PROTEIN DKFZP686K03196	unknown	unknown	o
IPI00433833	UNCHARACTERIZED PROTEIN (SIMILAR TO THO COMPLEX SUBUNIT 3)	unknown	unknown	x
IPI00445778	CDNA FLJ60358, HIGHLY SIMILAR TO REGULATOR OF G-PROTEIN SIGNALING 3	unknown	unknown	x
IPI00451401	ISOFORM 2 OF TRIOSEPHOSPHATE ISOMERASE	4, 5, 7, 8	12, 16	o
IPI00477003	COILED-COIL DOMAIN-CONTAINING PROTEIN KIAA1407	unknown	unknown	x
IPI00478115	ISOFORM 2 OF DELETED IN MALIGNANT BRAIN TUMORS 1 PROTEIN	1, 2, 3, 4, 6, 7	12, 15, 18	o
IPI00479295	GOLGIN SUBFAMILY A MEMBER 8-LIKE PROTEIN 2	unknown	unknown	x
IPI00514669	UNCHARACTERIZED PROTEIN (SIMILAR TO SH3 DOMAIN-BINDING GLUTAMIC ACID-RICH-LIKE PROTEIN 3)	unknown	unknown	x
IPI00556287	PUTATIVE UNCHARACTERIZED PROTEIN	unknown	unknown	x
IPI00556391	ACTIN-LIKE PROTEIN	unknown	unknown	x
IPI00641229	IG ALPHA-2 CHAIN C REGION	1, 2, 3, 4, 5, 6, 7, 10	13	x
IPI00643174	GATA-TYPE ZINC FINGER PROTEIN 1	4, 5, 7, 10	11, 12, 13, 19	x
IPI00644220	CDNA FLJ60139, HIGHLY SIMILAR TO HOMO SAPIENS HIV TAT SPECIFIC FACTOR 1 (HTATSF1), MRNA	unknown	unknown	x
IPI00646265	58 KDA PROTEIN (SIMILAR TO AMYLASE)	5	12, 13, 16	x
IPI00646771	ISOFORM 4 OF DISKS LARGE HOMOLOG 2	3, 5, 7, 10	12, 13, 16	x
IPI00657660	UNCHARACTERIZED PROTEIN (SIMILAR TO HEMOGLOBIN SUBUNIT DELTA)	6	13, 14	o
IPI00657911	UNCHARACTERIZED PROTEIN (SIMILAR TO HEMOGLOBIN SUBUNIT GAMMA-2)	6	13, 14	o
IPI00719452	IGL@ PROTEIN	unknown	unknown	x
IPI00738655	POTE ANKYRIN DOMAIN FAMILY MEMBER J	unknown	unknown	x
IPI00739539	POTE ANKYRIN DOMAIN FAMILY MEMBER F	unknown	unknown	o
IPI00740545	POTE ANKYRIN DOMAIN FAMILY MEMBER I	unknown	unknown	x
IPI00742996	RING FINGER PROTEIN 214	unknown	13	x
IPI00743194	KAPPA LIGHT CHAIN VARIABLE REGION	unknown	unknown	x
IPI00745468	SIMILAR TO TRANSMEMBRANE PROTEIN 90A	1	unknown	x
IPI00746516	PEJVAKIN	unknown	unknown	x
IPI00759479	ISOFORM 1 OF IQ MOTIF AND SEC7 DOMAIN-CONTAINING PROTEIN 2	3, 7	unknown	x
IPI00759832	ISOFORM SHORT OF 14-3-3 PROTEIN BETA/ALPHA	3, 4, 5, 6, 7, 10	12	o
IPI00761051	ISOFORM 1 OF ERYTHROID DIFFERENTIATION-RELATED FACTOR 1	4, 5, 10	12	x
IPI00783184	IMMUNGLOBULIN HEAVY CHAIN VARIABLE REGION	unknown	unknown	x
IPI00783287	IMMUNGLOBULIN HEAVY CHAIN VARIABLE REGION	unknown	unknown	x
IPI00783393	IMMUNGLOBULIN HEAVY CHAIN VARIABLE REGION	unknown	unknown	x
IPI00783909	IMMUNGLOBULIN HEAVY CHAIN VARIABLE REGION	unknown	unknown	x
IPI00794543	CDNA FLJ75174, HIGHLY SIMILAR TO HOMO SAPIENS CALMODULIN 1 (PHOSPHORYLASE KINASE, DELTA), MRNA	unknown	unknown	x
IPI00816314	PUTATIVE UNCHARACTERIZED PROTEIN DKFZP686I15196	unknown	unknown	o
IPI00816799	RHEUMATOID FACTOR D5 LIGHT CHAIN	unknown	unknown	x
IPI00827510	HRV FAB 026-VL	unknown	unknown	x
IPI00827560	HRV FAB N27-VL	unknown	unknown	x
IPI00827581	VARIABLE IMMNOGLOBULIN ANTI-ESTRADIOL HEAVY CHAIN	unknown	unknown	x
IPI00827618	VH6DJ PROTEIN	unknown	unknown	x
IPI00827690	VH-3 FAMILY (VH26)D/J PROTEIN	unknown	unknown	x
IPI00827789	HRV FAB N6-VL	unknown	unknown	x
IPI00827826	COLD AGGLUTININ FS-2 L-CHAIN	unknown	unknown	x
IPI00828061	ANTI-MUCIN1 HEAVY CHAIN VARIABLE REGION	unknown	unknown	x
IPI00828088	VH6DJ PROTEIN	unknown	unknown	x
IPI00828099	UGA8H	unknown	unknown	x
IPI00829590	13 KDA PROTEIN (SIMILAR TO IMMUNOGLOBULIN HEAVY VARIABLE 3–72)	unknown	unknown	x
IPI00829600	13 KDA PROTEIN (SIMILAR TO IMMUNOGLOBULIN KAPPA VAIRABLE 2D-26)	unknown	unknown	x
IPI00829701	13 KDA PROTEIN (SIMILAR TO IMMUNOGLOBULIN HEAVY VARIABLE 1–18)	unknown	unknown	x
IPI00829752	MYOSIN-REACTIVE IMMUNOGLOBULIN HEAVY CHAIN VARIABLE REGION	unknown	unknown	x
IPI00829812	13 KDA PROTEIN (SIMILAR TO IMMUNOGLOBULIN HEAVY VARIABLE 3–13)	3, 4, 6, 10	13, 16	x
IPI00829841	13 KDA PROTEIN (SIMILAR TO IMMUNOGLOBULIN HEAVY VARIABLE 3–73)	unknown	unknown	x
IPI00829845	IMMUNGLOBULIN HEAVY CHAIN VARIABLE REGION	unknown	unknown	o
IPI00829896	HEMOGLOBIN LEPORE-BALTIMORE	unknown	unknown	o
IPI00829956	RHEUMATOID FACTOR C6 LIGHT CHAIN	unknown	unknown	x
IPI00844168	INTERFERON-INDUCIBLE PROTEIN AIM2	1, 2, 3, 4, 5, 6, 7, 10	11, 12	x
IPI00844239	IMMUNOBLOBULIN G1 FAB HEAVY CHAIN VARIABLE REGION	unknown	unknown	x
IPI00847618	UNCHARACTERIZED PROTEIN (SIMILAR TO SH3 AND MULTIPLE ANKYRIN REPEAT DOMAINS PROTEIN 3)	1, 3, 4, 6, 7, 9, 10	12, 13, 18	x
IPI00853045	ANTI-RHD MONOCLONAL T125 KAPPA LIGHT CHAIN	unknown	unknown	x
IPI00853455	PROTEIN	unknown	unknown	x
IPI00853525	UNCHARACTERIZED PROTEIN (Apolipoprotein A)	4, 6	13	o
IPI00853641	UNCHARACTERIZED PROTEIN (SIMILAR TO HEMOGLOBIN SUBUNIT EPSILON)	6	13, 14	x
IPI00854644	IG KAPPA CHAIN V-III REGION TI	3, 4, 6, 10	13, 16	x
IPI00854732	SIMILAR TO MYOSIN-REACTIVE IMMUNOGLOBULIN HEAVY CHAIN VARIABLE REGION	unknown	unknown	x
IPI00854743	IMMUNGLOBULIN HEAVY CHAIN VARIABLE REGION	unknown	unknown	x
IPI00854841	MYOSIN-REACTIVE IMMUNOGLOBULIN HEAVY CHAIN VARIABLE REGION	unknown	unknown	x
IPI00871622	PUTATIVE ZINC-ALPHA-2-GLYCOPROTEIN-LIKE 1	unknown	unknown	x
IPI00871681	PUTATIVE UNCHARACTERIZED PROTEIN ENSP00000344348	unknown	unknown	x
IPI00878173	CDNA FLJ39583 FIS, CLONE SKMUS2004897, HIGHLY SIMILAR TO ACTIN, ALPHA SKELETAL MUSCLE	unknown	unknown	x
IPI00878282	23 KDA PROTEIN (SIMILAR TO SERUM ALBUMIN)	6	unknown	o
IPI00884092	ANTI-HER3 SCFV	unknown	unknown	x
IPI00888712	PUTATIVE BETA-ACTIN-LIKE PROTEIN 3	6	11	x
IPI00888954	IMMUNGLOBULIN HEAVY CHAIN VARIABLE REGION	unknown	unknown	x
IPI00890703	CRYOCRYSTALGLOBULIN CC1 KAPPA LIGHT CHAIN VARIABLE REGION	unknown	unknown	x
IPI00890754	CRYOCRYSTALGLOBULIN CC1 HEAVY CHAIN VARIABLE REGION	unknown	unknown	x
IPI00892724	CELL DIVISION CYCLE-ASSOCIATED 7-LIKE PROTEIN ISOFORM 2	unknown	unknown	o
IPI00893862	SFI1 HOMOLOG, SPINDLE ASSEMBLY ASSOCIATED	unknown	unknown	x
IPI00894523	UNCHARACTERIZED PROTEIN (SIMILAR TO ACTIN, CYTOPLASMIC 1)	unknown	unknown	o
IPI00896380	ISOFORM 2 OF IG MU CHAIN C REGION	1, 2, 3, 4, 6, 7, 10	11, 12, 13	x
IPI00903112	CDNA FLJ36533 FIS, CLONE TRACH2004428, HIGHLY SIMILAR TO LACTOTRANSFERRIN	unknown	unknown	x
IPI00908653	CDNA FLJ60121, HIGHLY SIMILAR TO UNC-13 HOMOLOG B	unknown	unknown	x
IPI00909257	CDNA FLJ50149, HIGHLY SIMILAR TO HOMO SAPIENS ATTRACTIN-LIKE 1 (ATRNL1), MRNA	unknown	unknown	x
IPI00909649	IG KAPPA CHAIN C REGION	1, 2, 3, 4, 6, 7, 10	13, 16	x
IPI00910380	CDNA FLJ54278, HIGHLY SIMILAR TO SPARC-LIKE PROTEIN 1	unknown	unknown	x
IPI00910779	CDNA FLJ52141, HIGHLY SIMILAR TO 14-3-3 PROTEIN GAMMA	unknown	unknown	x
IPI00915869	MALATE DEHYDROGENASE, CYTOPLASMIC ISOFORM 3	5	16	x
IPI00916434	ANTI-(ED-B) SCFV	unknown	unknown	x
IPI00921079	CDNA FLJ61647, HIGHLY SIMILAR TO NUCLEOLAR COMPLEX PROTEIN 2 HOMOLOG	unknown	unknown	o
IPI00922693	CDNA FLJ53662, HIGHLY SIMILAR TO ACTIN, ALPHA SKELETAL MUSCLE	unknown	unknown	o
IPI00924681	UNCHARACTERIZED PROTEIN (SIMILAR TO ZINC FINGER PROTEIN 568)	unknown	unknown	x
IPI00924820	SIMILAR TO KAPPA LIGHT CHAIN VARIABLE REGION	unknown	unknown	x
IPI00924948	UNCHARACTERIZED PROTEIN (SIMILAR TO ZINC-ALPHA-2-GLYCOPROTEIN)	unknown	13	o
IPI00925547	UNCHARACTERIZED PROTEIN (SIMILAR TO LACTOTRANSFERRIN)	1, 2, 4, 10	13, 16	x
IPI00930072	PUTATIVE UNCHARACTERIZED PROTEIN DKFZP686E23209	unknown	unknown	x
IPI00930124	PUTATIVE UNCHARACTERIZED PROTEIN DKFZP686C11235	unknown	unknown	o
IPI00930351	HBBM FUSED GLOBIN PROTEIN	unknown	unknown	o
IPI00930404	ISOFORM 2 OF KALLIKREIN-1	4	16	o
IPI00930442	PUTATIVE UNCHARACTERIZED PROTEIN DKFZP686M24218	1, 2, 3, 4, 6, 7, 10	13, 16	x
IPI00937669	10 KDA PROTEIN	unknown	unknown	x
IPI00939278	UNCHARACTERIZED PROTEIN (SIMILAR TO ZINC-ALPHA-2-GLYCOPROTEIN)	unknown	13	o
IPI00940245	IMMUNOGLOBULIN HEAVY CHAIN VARIANT	unknown	unknown	x
IPI00940727	10 KDA PROTEIN	unknown	unknown	x
IPI00943106	UNCHARACTERIZED PROTEIN (SIMILAR TO GOLGIN SUBFAMILY A MEMBER 8N)	unknown	unknown	x
IPI00953352	ISOFORM 10 OF PROTEIN SFI1 HOMOLOG	5, 10	12	x
IPI00956140	HCG1652138, ISOFORM CRA_A	unknown	unknown	x
IPI00956602	ANTI-STREPTOCOCCAL/ANTI-MYOSIN IMMUNOGLOBULIN LAMBDA LIGHT CHAIN VARIABLE REGION	unknown	unknown	x
IPI00964049	UNCHARACTERIZED PROTEIN (SIMILAR TO SPECKL-TYPE POZ PROTEIN)	unknown	unknown	x
IPI00965653	12 KDA PROTEIN (SIMILAR TO IMMUNOGLOBULIN KAPPA VARIABLE 2D-40)	3, 4, 6, 10	13, 16	x
IPI00966829	69 KDA PROTEIN (SIMILAR TO SERUM ALBUMIN)	6	unknown	o
IPI00966961	PROTEIN (SIMILAR TO CENTROSOMAL PROTEIN 192 KDA)	7, 10	unknown	x
IPI00969456	IGKC PUTATIVE UNCHARACTERIZED PROTEIN	unknown	unknown	x
IPI00969620	LIGHT CHAIN FAB	unknown	unknown	x
IPI00972963	LAMBDA LIGHT CHAIN OF HUMAN IMMUNOGLOBULIN SURFACE ANTIGEN-RELATED PROTEIN	unknown	unknown	x
IPI00973016	UBIQUITOUSLY TRANSCRIBED TETRATRICOPEPTIDE REPEAT PROTEIN Y-LINKED TRANSCRIPT VARIANT 27	unknown	unknown	x
IPI00973032	V1-17 PROTEIN	unknown	unknown	x
IPI00973424	IGLC1 PUTATIVE UNCHARACTERIZED PROTEIN	unknown	unknown	x
IPI00973474	PUTATIVE UNCHARACTERIZED PROTEIN (SIMILAR TO IG GAMMA-3 CHAIN C REGION)	1, 2, 3, 4, 6, 7, 10	13, 16	o
IPI00973513	IMUNOGLOBULIN HEAVY CHAIN	unknown	unknown	x
IPI00973531	IGLC2 PUTATIVE UNCHARACTERIZED PROTEIN	unknown	unknown	x
IPI00974544	ISOFORM SV OF 14-3-3 PROTEIN EPSILON	1, 2, 3, 4, 6, 7, 10	12	o
IPI00976299	UNCHARACTERIZED PROTEIN (SIMILAR TO UTEROGLOBIN)	3	unknown	o
IPI00976559	UNCHARACTERIZED PROTEIN (SIMILAR TO DIPHTHAMIDE BIOSYNTHESIS PROTEIN 2)	4	unknown	x
IPI00976928	SIMILAR TO MYOSIN-REACTIVE IMMUNOGLOBULIN HEAVY CHAIN VARIABLE REGION	unknown	unknown	x
IPI00977221	HYPOTHETICAL PROTEIN LOC100291917	unknown	unknown	x
IPI00977405	SIMILAR TO IG KAPPA CHAIN V-III REGION VG PRECURSOR	3, 4, 6, 10	13, 16	x
IPI00977733	SIMILAR TO VH-7 FAMILY (N54P3)D/J PROTEIN	unknown	unknown	x
IPI00977788	SIMILAR TO HEPATITIS B VIRUS RECEPTOR BINDING PROTEIN	unknown	unknown	x
IPI00978208	PEPTIDYL-PROLYL CIS-TRANS ISOMERASE A-LIKE	4, 5, 6, 7, 10	12, 13, 16	o
IPI00978930	IG ALPHA-1 CHAIN C REGION	1, 2, 3, 4, 5, 6, 7, 10	13	x
IPI00979730	SIMILAR TO IG KAPPA CHAIN V-II REGION GM607 PRECURSOR	3, 4, 6, 10	13, 16	x
IPI00980227	CONSERVED HYPOTHETICAL PROTEIN MUC5B	1, 2, 4, 10	12	x
IPI00981659	SIMILAR TO COLD AGGLUTININ FS-1 H-CHAIN	unknown	unknown	x
IPI00982101	UNCHARACTERIZED PROTEIN	unknown	12	o
IPI00983257	NEURONAL ACETYLCHOLINE RECEPTOR ALPHA-4 SUBUNIT	unknown	unknown	x
IPI00983475	SIMILAR TO HEPATITIS B VIRUS RECEPTOR BINDING PROTEIN	unknown	unknown	x
IPI00985150	11 KDA PROTEIN	unknown	unknown	x
IPI00985211	SIMILAR TO VH-3 FAMILY (VH26)D/J PROTEIN	unknown	unknown	x
IPI00985251	PROTEIN (SIMILAR TO UPF0317 PROTEIN C14ORF159)	unknown	unknown	x
IPI01010056	58 KDA PROTEIN (SIMILAR TO AMYLASE)	5	12, 13, 16	x
IPI01011189	MUC5B UNCHARACTERIZED PROTEIN	unknown	unknown	x
IPI01011784	UNCHARACTERIZED PROTEIN (SIMILAR TO E3 UBIQUITIN-PROTEIN LIGASE MDM2)	unknown	unknown	o
IPI01012504	6-PHOSPHOGLUCONATE DEHYDROGENASE, DECARBOXYLATING	4, 5	16	o
IPI01013214	RHEUMATOID FACTOR RF-IP14	unknown	unknown	x
IPI01014621	UNCHARACTERIZED PROTEIN (SIMILAR TO ZINC FINGER PROTEIN 705A)	4, 5, 10	11	x
IPI01014804	UNCHARACTERIZED PROTEIN	unknown	unknown	x
IPI01015266	PUTATIVE UNCHARACTERIZED PROTEIN DKFZP686O16217	unknown	unknown	x
IPI01017938	IG LAMBDA-6 CHAIN C REGION	1, 2, 3, 4, 6, 7, 10	13, 16	x
IPI01018060	IG LAMBDA-3 CHAIN C REGIONS	1, 2, 3, 4, 6, 7, 10	13, 16	x
IPI01018161	PYRUVATE KINASE ISOZYMES M1/M2 ISOFORM C	1, 2, 4, 5, 7, 8, 10	11, 12, 13, 16	o
IPI01018257	HYPOTHETICAL PROTEIN (SIMILAR TO POLYMERIC IMMUNOGLOBULIN RECEPTOR)	unknown	unknown	o
IPI01018712	ACTA2 PROTEIN	unknown	unknown	x
IPI01018716	TOSPEAK-5	unknown	unknown	x
IPI01018887	UBIQUITOUSLY TRANSCRIBED TETRATRICOPEPTIDE REPEAT PROTEIN Y-LINKED TRANSCRIPT VARIANT 8	unknown	unknown	x
IPI01018897	MUCIN-5AC	unknown	unknown	o
IPI01018949	CDNA FLJ51266, HIGHLY SIMILAR TO VITRONECTIN	unknown	unknown	x
IPI01022126	UNCHARACTERIZED PROTEIN (SIMILAR TO KAT8 REGULATORY NSL COMPLEX SUBUNIT 2)	unknown	unknown	x
IPI01022319	UNCHARACTERIZED PROTEIN (SIMILAR TO TWINFILIN-1)	unknown	unknown	x
IPI01022662	UNCHARACTERIZED PROTEIN (SIMILAR TO CARBONIC ANHYDRASE 6)	5	13, 16	x
IPI01022818	15 KDA PROTEIN (SIMILAR TO LYSOZYME)	1, 2, 4	unknown	o
IPI01022820	AMYLOID LAMBDA 6 LIGHT CHAIN VARIABLE REGION SAR	unknown	unknown	x
IPI01024840	PROTEIN (SIMILAR TO X-LINKED RETINITIS PIGMENTOSA GTPASE REGULATOR-INTERACTING PROTEIN 1)	7	unknown	x
IPI01025882	MYOSIN-REACTIVE IMMUNOGLOBULIN LIGHT CHAIN VARIABLE REGION	unknown	unknown	x
IPI01026053	PROTEIN (SIMILAR TO EPIDIDYMAL SECRETORY PROTEIN E1)	unknown	unknown	x

For the determination of the inter-platform variability in the nLC-Q-IMS-TOF system used in this study and the validation of the identities of proteins, especially, the distinct Korean WS proteins, results of the analyses of the pooled Korean WS sample by the nLC-Q-IMS-TOF system and a nLC-Q-orbitrap system were compared. As a result, 141 and 208 proteins were identified from the nLC-Q-TOF platform and the nLC-Q-orbitrap platform, respectively, and 98 out of 141 proteins (69.5%) from the nLC-Q-TOF platform were overlapped with those from the nLC-Q-orbitrap platform. Among proteins identified in the pooled sample, 130 proteins from the nLC-Q-TOF platform and 147 proteins from the nLC-Q-orbitrap platform were found to be within the Korean WS proteome index. Additionally, among those proteins overlapped with the Korean WS proteome, 22 out of 130 proteins (16.9%) and 29 out of 147 proteins (19.7%) were confirmed to belong to the distinct Korean WS proteins from the nLC-Q-TOF platform and the nLC-Q-orbitrap platform, respectively. Finally, the portion of the distinct proteins from the nLC-Q-IMS-TOF platform, which overlaps with those from the nLC-Q-orbitrap plarform was 68.2% (S3 and S4 Tables and S1 Fig).

**Fig 3:**
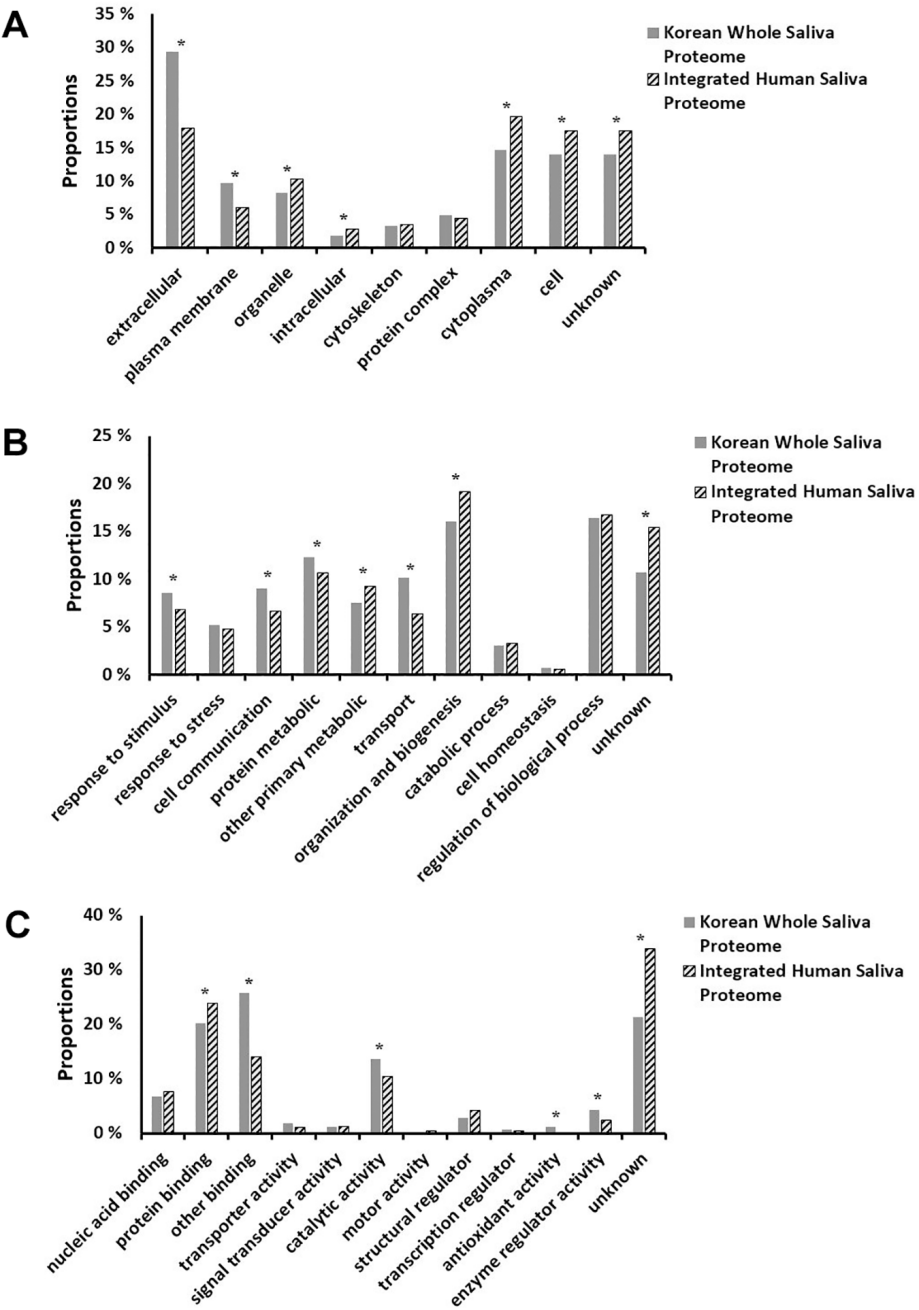
**Relative allocation and comparison of proteins observed in the Korean whole saliva proteome and the integrated human saliva proteome according to their gene ontology annotations in terms of cellular component (A), biological process (B), and molecular function (C).** *, *p* < 0.05.

In addition to the comparison of protein identities, GO annotation in terms of cellular component, biological process, and molecular function between the Korean WS proteome and the integrated human saliva proteome was compared (Fig 3). First, in GO cellular component categories, the Korean WS proteome was significantly over-represented in extracellular space and the plasma membrane but under-represented in organelle, intracellular, cytoplasma, and the cell compared to the integrated human saliva proteome (*p* < 0.05). GO biologic process categories also showed higher portions of proteins for response to stimulus, cell communication, protein metabolism, and transport in the Korean WS proteome than those in the integrated human saliva proteome (*p* < 0.05). However, the opposite tendency was observed in proteins for other primary metabolic and organization and biogenesis (*p* < 0.05). Finally, in the case of GO molecular function categories, over-representation of the Korean WS proteome was observed in other binding, catalytic activity, antioxidant activity, and enzyme regulatory activity with under-representation in protein binding compared to the integrated human saliva proteome were found (*p* < 0.05). Allocation of proteins observed in the Korean WS proteome according to their GO annotation can be found in S5–S7 Tables.

### Distinct Korean WS proteins and diseases

To evaluate the clinical applicability of ethnicity-specific human saliva proteome, 226 proteins observed in the Korean WS proteome, but not in the integrated human saliva proteome, were searched against the Database of disease-related biomarkers [24]. As shown in Table 1 and S2 Table, 50 of 226 distinct Korean WS proteins (22.1%) were found to be disease biomarker candidates. Also, Table 2 and S2 Table indicate that 7–21 of these distinct Korean WS proteins are probably associated with individual conditions representing the top 10 deadliest diseases in South Korea, 2015 (cerebrovascular disease, lung cancer, ischemic heart disease, liver cancer, diabetes mellitus, stomach cancer, colorectal cancer, pancreatic cancer, hypertension, and dementia) [25].

**Table 2:** Distinct Korean WS proteins probably associated with the top 10 deadliest diseases in South Korea, 2015.

Rank	Disease (A)	The distinct Korean whole saliva proteins probably associated with A
1	Cerebrovascular disease	IPI00000149, IPI00003842, IPI00022434, IPI00216773, IPI00878282, IPI00966829, IPI00150554, IPI00892724, IPI00218573, IPI00220644, IPI01018161, IPI00305457, IPI00853525, IPI00930404
2	Lung cancer	IPI00000149, IPI00003842, IPI00020019, IPI00022434, IPI00216773, IPI00878282, IPI00966829, IPI00103636, IPI00150554, IPI00892724, IPI00220644, IPI01018161, IPI00376170, IPI00978208, IPI00478115, IPI00976299, IPI01018257, IPI01011784, IPI01018897, IPI01022818, IPI00930404
3	Ischemic heart disease	IPI00000149, IPI00020019, IPI00022434, IPI00216773, IPI00878282, IPI00966829, IPI00103636, IPI00150554, IPI00892724, IPI00218573, IPI00305457, IPI00376170, IPI00978208, IPI00411765, IPI00451401, IPI00739539, IPI00853525, IPI01011784, IPI00930404
4	Liver cancer	IPI00000149, IPI00022434, IPI00216773, IPI00878282, IPI00966829, IPI00150554, IPI00892724, IPI00181753, IPI00220644, IPI01018161, IPI00305457, IPI00451401, IPI00739539, IPI00853525, IPI01018257, IPI01011784, IPI01018897, IPI01022818
5	Diabetes mellitus	IPI00000149, IPI00018214, IPI00020019, IPI00022434, IPI00216773, IPI00878282, IPI00966829, IPI00150554, IPI00892724, IPI00305457, IPI00853525, IPI00976299, IPI00930404
6	Stomach cancer	IPI00022434, IPI00216773, IPI00878282, IPI00966829, IPI00305457, IPI01018897, IPI01022818
7	Colorectal cancer	IPI00000149, IPI00018214, IPI00019226, IPI00022434, IPI00216773, IPI00878282, IPI00966829, IPI00026126, IPI00150554, IPI00892724, IPI00220644, IPI01018161, IPI00739539, IPI01018257, IPI01011784, IPI01018897, IPI01022818
8	Pancreatic cancer	IPI00022434, IPI00216773, IPI00878282, IPI00966829, IPI00103636, IPI00220644, IPI01018161, IPI00411765, IPI01018897, IPI01022818, IPI00930404
9	Hypertension	IPI00018214, IPI00022434, IPI00216773, IPI00878282, IPI00966829, IPI00150554, IPI00892724, IPI00218573, IPI00657911, IPI00853525, IPI00976299, IPI01022818, IPI00930404
10	Dementia	IPI00000149, IPI00003842, IPI00022434, IPI00216773, IPI00878282, IPI00966829, IPI00150554, IPI00892724, IPI00181753, IPI00218573, IPI00220644, IPI01018161, IPI00305457, IPI00376170, IPI00978208, IPI00739539, IPI00853525, IPI00924948, IPI00939278, IPI00930351, IPI00930404

## Discussion

As the initial step to determine ethnic difference of human saliva proteome, the Korean WS proteome was constructed for the first time due to the fact that Korea is the most ethnically homogenous country in the world [26]. A total of 480 proteins are catalogued in the Korean WS proteome (S1 Table), including most of commonly observed saliva proteins (amylase, cystantins, acidic proline rich proteins, basic proline rich proteins, mucins, lactotransferrin, carbonic anhydrase, lysozymes, peroxidases, albumin, and statherines) [11, 27]. This observation indicated that the analytical method employed in the present study was performed properly. However, three groups of common saliva proteins (thymosins, defensins, and histatins) were not observed in the present study. Although an exact explanation on their absence cannot be provided with certainty, loss during sample preparation, the under-sampling issue of mass spectrometry brought by the complexity of a sample, their cleavage into small peptides, and/or binding of the resulting peptides to tissues may contribute to their absence [11, 27, 28].

For the actual determination of ethnic differences in human saliva proteome, the present Korean WS protein list was compared to the integrated human saliva protein list in a couple of ways. First, comparison of protein identities in each list revealed that 47.1% (226 out of 480) of proteins were unique in the Korean WS proteome. Discovering a large portion of Korean WS unique proteins from the Korean WS proteome was expected, because similar portion to that (54.1%, 100 out of 185 proteins) was already reported from distinct Korean plasma proteins compared to human plasma proteome [18]. However, there is a possibility of identifying common proteins for the first time by employing different analytical techniques, which would weaken the possibility of the connection between the distinct Korean WS proteins and ethnic differences in human saliva proteome. Thus, for the determination of the inter-platform variability in the nLC-Q-IMS-TOF system used in this study and the validation of the identities of proteins (especially, the distinct Korean WS proteins) simultaneously, results of the analyses of the pooled Korean WS sample by the nLC-Q-IMS-TOF system and a nLC-Q-orbitrap system, a platform widely used for proteomics were compared. As a result, 141 and 208 proteins were identified from the nLC-Q-TOF platform and the nLC-Q-orbitrap platform, respectively, and 98 out of 141 proteins (69.5%) from the nLC-Q-TOF platform were overlapped with those from the nLC-Q-orbitrap platform (S3 and S4 Tables and S1 Fig). If about 70–80% of the repeatability (the inner-system comparison) and about 60–80% of the reproducibility (the inter-system including inter-platform comparison) of a standardized analysis platform in protein identification are considered [29], no significant influence of the inter-platform variability in the nLC-Q-IMS-TOF system as well as the high credibility of protein identities in the Korean WS proteome can be urged. Among proteins identified in the pooled sample, 130 proteins from the nLC-Q-TOF platform and 147 proteins from the nLC-Q-orbitrap platform were found to be within the Korean WS proteome index. Additionally, among those proteins overlapped with the Korean WS proteome, 22 out of 130 proteins (16.9%) and 29 out of 147 proteins (19.7%) were confirmed to belong to the distinct Korean WS proteins from the nLC-Q-TOF platform and the nLC-Q-orbitrap platform, respectively (S3 and S4 Tables and S1 Fig). *The numbers of every type of proteins identified from the analyses of the pooled sample by using each platform are smaller than the counter parts of the Korean WS proteome*, likely the consequence of the dilution of individual proteins by saliva pooling and the analyses of a single sample. However, the portion of the distinct proteins from the nLC-Q-IMS-TOF platform, which overlaps with those from the nLC-Q-orbitrap platform, is still close to 70% (68.2%). Thus, no significant influence of the inter-platform variability in our system is observed, lending high credibility of the protein identities in the Korean WS proteome. Interestingly, the number of proteins from the nLC-Q-orbitrap platform, which belong to distinct protein in Korean WS is larger than that from the nLC-Q-IMS-TOF platform (22 proteins from the nLC-Q-IMS-TOF platform *vs*. 29 proteins from the nLC-Q-orbitrap platform; S3 and S4 Tables and S1 Fig). This observation provides additional evidence to support the high credibility of the identities of the distinct Korean WS proteins. Therefore, since the existence of the distinct Korean WS proteins are more confident and the possibility of their identification by the inter-platform variability can be significantly excluded, ethnic differences in the human saliva proteome, especially in the Korean WS proteome become more evident.

While it was observed that the nLC-Q-IMS-TOF system of this study did not bring higher performance than other proteomics platforms due to the relatively small number of proteins identified, the identification of the distinct proteins confirmed that the nLC-Q-IMS-TOF system still has good performance for the identification of distinct Korean WS proteins. Actually, to build a global protein list, most proteomic studies have employed multi-dimensional proteomics technique to include as many as possible proteins in their lists [1, 7–11, 30, 31]. However, such multi-dimensional proteomics technique demands enormous analysis time and computing power for protein identification. Therefore, we chose the combination of nLC-Q-TOF (a single dimensional technique) and IMS (an additional technique to separate ions based on their different mobility in a carrier gas) “on-line” instead of using the conventional multi-dimensional technique [28, 32]. To the best of our knowledge, this is the first study that applies IMS to saliva proteomics.

From comparison of GO annotations between the Korean WS proteome and the integrated human saliva proteome, some categories in the Korean WS proteome showed over-representation or under-representation (Fig 3). Regarding their applications to biomarker-related studies, over-represented categories might be more important than under-represented ones due to the probability of finding more meaningful information from more proteins belonging to over-represented categories. In the present study, over-represented GO categories in the Korean WS proteome are as follows: extracellular and plasma membrane of cellular components (Fig 3A), response to stimulus, cell communication, protein metabolic, and transport of biological processes (Fig 3B), and other binding, catalytic activity, antioxidant activity, and enzyme regulatory activity of molecular function (Fig 3C). Interestingly, most of them can provide substantial information on diseases due to their connectivity to disease-related features such as extracellular secretion for biological function (the extracellular category of cellular component), the defense system of the body (the response to stimulus category of biological processes), cellular signal transduction (the cell communication category of biological processes), chemical reactions and pathways involving a specific proteins (the protein metabolic category of biological processes), positioning of a substance or cellular entity (the transport category of biological processes), non-covalent interaction of a non-protein molecule with specific site(s) on another molecule (the other binding category of the biological processes), catalysis of a biochemical reaction (the catalytic activity category of the biological processes), inhibition of oxidation (the antioxidant activity category of the biological processes), and/or modulation (by direct binding) of the activity of an enzyme (enzyme regulator activity category for molecular function) [1, 2, 10, 33]. Additionally, over-representation of protein metabolic and catalytic activity categories in the Korean WS proteome compared with the integrated human saliva proteome may be consistent with its larger portion of proteins with molecular weight of less than 60 kDa (82.3%, Fig 1A), partially resulting from the cleavage of higher-molecular-weight proteins, than that of Yan et al.'s report (68%) [1]. In line with these findings, our results suggest another clue to discover ethnic differences in the human saliva proteome and the possibility of using such difference for early diagnosis and/or prognosis of diseases.

For further evaluation of the clinical applicability of ethnicity-specific human saliva proteome, 226 distinct proteins observed in Korean WS, but not in other human saliva, were searched through the Database of disease-related biomarkers. As a result, 22.1% (50 out of 226) of these distinct proteins were found to be disease biomarker candidates (Table 1 and S2 Table), firmly supporting the probable value of using ethnicity-specific human saliva proteome for disease biomarker applications. Also, all top 10 deadliest diseases in South Korea, 2015 (cerebrovascular disease, lung cancer, ischemic heart disease, liver cancer, diabetes mellitus, stomach cancer, colorectal cancer, pancreatic cancer, hypertension, and dementia) are found to have at least 7 disease biomarker candidates which belong to the distinct Korean WS proteins (Table 2 and S2 Table) [25]. The total number of distinct Korean WS proteins probably associated with the top 10 deadliest diseases in South Korea is 35, representing 70.0% (35 out of 50) of disease biomarker candidate proteins among distinct Korean WS proteins (Tables 1 and 2 and S2 Table). Thus, this result clearly shows that ethnicity-specific human saliva proteins have diagnostic potential for diseases highly prevalent in that ethnic group.

However, this study has a couple of limitations. First, as mentioned above, it did not employ any multi-dimensional separation technique, and, as a result, a relatively small number of proteins was catalogued in the Korean WS proteome index. Interestingly, however, its limited performance must have played an important role in supporting ethnicity-related differences in human saliva, because it did not seem to produce any significant platform-specific performance, the source of the inter-platform variability. Also, since WS samples were collected from only eleven young male adult volunteers, there would be concerns of gender bias as well as a lack of representativeness in the results because of the narrow age range of the participants. Thus, the expansion of the Korean WS proteome by analyzing more samples, including female WS and a broader range of participant ages, by using the nLC-Q-IMS-TOF system or a multi-dimensional proteomics technique is expected in the near future.

## Conclusions

The Korean WS proteome catalogue indexing 480 proteins was built and characterized from nLC-Q-IMS-TOF analyses of WS samples collected from eleven healthy Korean male adult volunteers in this study for the first time. From comparison of the Korean WS proteome with the integrated human saliva proteome in terms of protein identities and GO annotations, evidences strongly support ethnic difference in human saliva proteome. Additionally, the potential value of ethnicity-specific human saliva proteins as biomarkers for diseases highly prevalent in that ethnic group was confirmed by finding 35 distinct Korean WS proteins probably associated with the top 10 deadliest diseases in South Korea. Finally, the present Korean WS protein list can serve as the first level reference for future proteomic studies including disease biomarker studies on Korean saliva.

## Supporting information

S1 Table A total of 480 Korean whole saliva proteins identified in the present study. Among multiple results on a certain protein from different sample and replicate analyses, only one with the highest PLGS score was selected for this table.
(XLSX)

S2 Table Distinct proteins observed in Korean whole saliva but not in other human saliva and their probable association with diseases including the top 10 deadliest diseases in South Korea, 2015. (XLSX)

S3 Table Proteins identified from the nLC-Q-IMS-TOF analysis of pooled Korean whole saliva. (XLSX)

S4 Table Proteins identified from the nLC-Q-orbitrap analysis of pooled Korean whole saliva. (XLSX)

S5 Table Allocation of proteins observed in the Korean whole saliva proteome according to their gene ontology annotation in terms of cellular component. (XLSX)

S6 Table Allocation of proteins observed in the Korean whole saliva proteome according to their gene ontology annotation in terms of biological process. (XLSX)

S7 Table Allocation of proteins observed in the Korean whole saliva proteome according to their gene ontology annotation in terms of molecular function. (XLSX)

S1 Fig Venn diagrams illustrating the number of proteins specific to either the nLC-Q-IMS-TOF analysis of pooled Korean whole saliva or the nLC-Q-orbitrap analysis of pooled Korean whole saliva proteome and those observed in both proteomes. Total proteins identified (A). Proteins which belong to the Korean whole saliva proteome (B). Proteins which belong to the distinct Korean whole saliva proteins (C).
(PPTX)

S1 Appendix Supplementary materials and methods. (DOCX)
