# Supplementary material for: Construction and characterization of the Korean whole saliva proteome to determine ethnic differences in human saliva proteome
Source: PLoS One. 2017 Jul 24;12(7):e0181765. doi: 10.1371/journal.pone.0181765 (PMC5524414; doi:10.1371/journal.pone.0181765)
Supplement: S1 Fig — Total proteins identified (A). Proteins which belong to the Korean whole saliva proteome (B). Proteins which belong to the distinct Korean whole saliva proteins (C). (PPTX) [file pone.0181765.s008.pptx]

## Slide 1
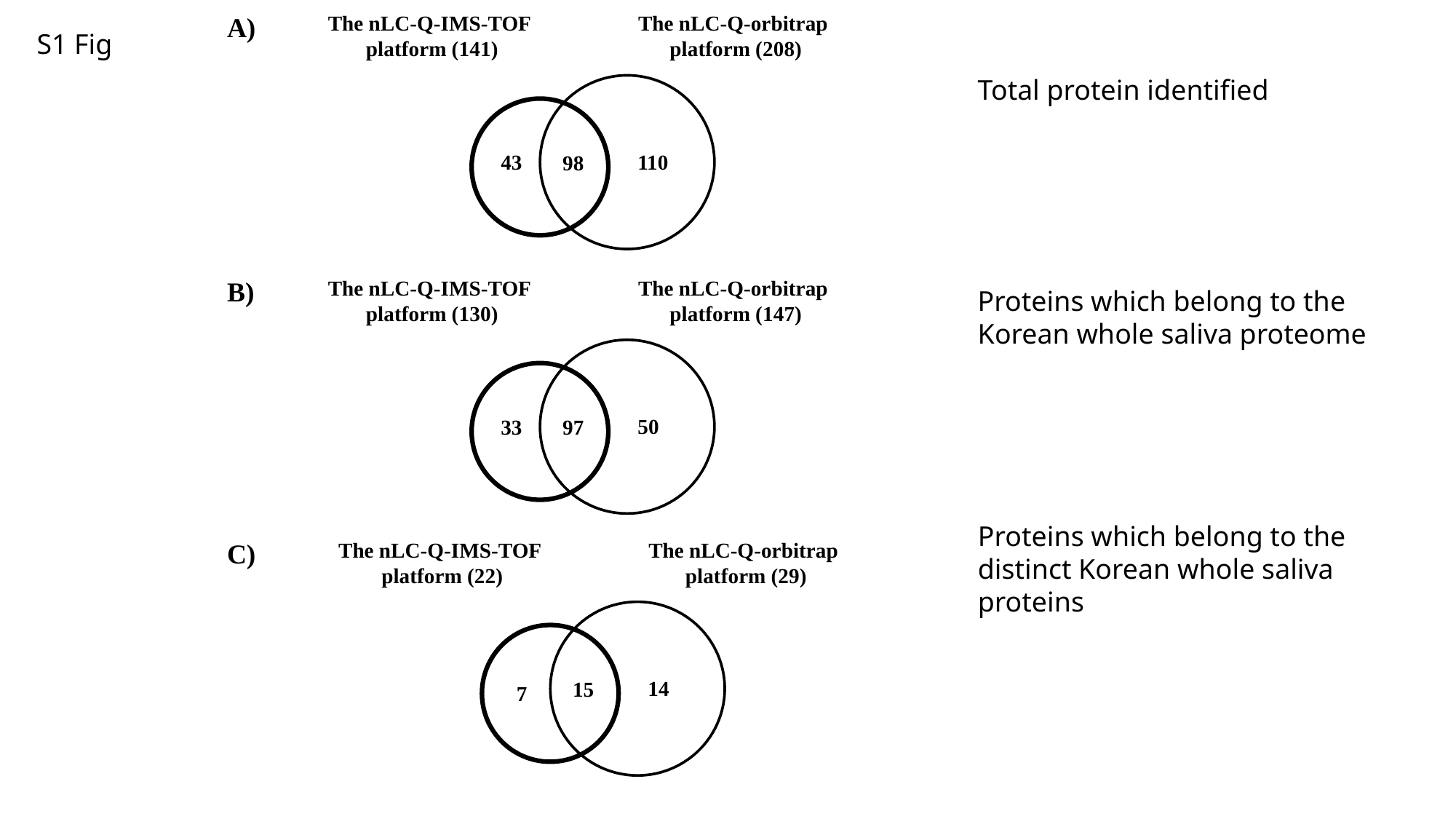

The nLC-Q-IMS-TOF
platform (141)
The nLC-Q-orbitrap
platform (208)
110
43
98
A)
S1 Fig
Total protein identified
B)
The nLC-Q-IMS-TOF
platform (130)
The nLC-Q-orbitrap
platform (147)
50
33
97
Proteins which belong to the
Korean whole saliva proteome
Proteins which belong to the distinct Korean whole saliva proteins
C)
The nLC-Q-IMS-TOF
platform (22)
The nLC-Q-orbitrap
platform (29)
14
15
7
