# Supplementary material for: Construction and characterization of the Korean whole saliva proteome to determine ethnic differences in human saliva proteome
Source: PLoS One. 2017 Jul 24;12(7):e0181765. doi: 10.1371/journal.pone.0181765 (PMC5524414; doi:10.1371/journal.pone.0181765)
Supplement: S1 Appendix — (DOCX) [file pone.0181765.s009.docx]

Construction and characterization of the Korean whole saliva proteome to determine ethnic differences in human saliva proteome

Ha Ra Cho^1^, Han Sol Kim^1^, Jun Seo Park^1^, Seung Cheol Park^2^, Kwang Pyo Kim^2^, Troy D. Wood^3*^, Yong Seok Choi^1*^

^1^ College of Pharmacy, Dankook University, Cheonan, Chungnam, South Korea

^2^ Department of Applied Chemistry, The Institute of National Science, College of Applied Science, Kyung Hee University, Yongin, Kyoungki, South Korea

^3^ Department of Chemistry, The State University of New York at Buffalo, Buffalo, New York, The United States of America

* Corresponding authors

E-mail: [twood@buffalo.edu](mailto:twood@buffalo.edu) (TDW)

[analysc@dankook.ac.kr](mailto:analysc@dankook.ac.kr) (YSC)

**Supplementary materials and methods**

Additional Procedures of Sample Digestion

The dried residue equivalent to 1 mg of total protein was resuspended, denatured, and reduced with 100 µL of 6 mol/L urea (in 0.1 mol/L Tris-HCl) and 5 µL of 0.2 mol/L dithiothreitol (in 0.1 mol/L Tris-HCl) at room temperature (RT) for one hour. Reduced protein in the mixture was then alkylated with 20 µL of 0.2 mol/L iodoacetamide (in 0.1 mol/L Tris-HCl) at RT for one hour in the dark. The alkylating reagent remaining in the resulting solution was quenched with 20 µL of 0.2 mol/L dithiothreitol (in 0.1 mol/L Tris-HCl) at RT for an hour. For protein digestion, 775 µL of water and 100 µL of 200 ng/µL trypsin (Promega, Madison, WI) dissolved in 0.1 mol/L Tris-HCl were then added and followed by incubation at 37ºC for 12 hours. The digestion was terminated by adding 1 µL of formic acid. The tryptic digest solution was dried by vacuum centrifugation and redissolved in 1 mL of 0.1% formic acid. The redissolved digest solution was diluted 8 times with 0.1% formic acid. A portion of the supernatant was subjected to nanoliquid chromatography-quadrupole-ion mobility spectroscopy-time of flight (nLC-Q-IMS-TOF) analysis.

In the case of the pooled Korean WS sample, 1 mL of each thawed protease-spiked sample supernatant was mixed and the mixture was applied to the same method mentioned above. A portion of the final form of the pooled sample solution was subjected to nLC-Q-IMS-TOF analysis and nLC-Q-orbitrap analysis.

Additional Procedures and Parameters of Separation and Analysis

In the case of nLC-Q-IMS-TOF analysis, peptides eluted from the analytical column of the nLC were delivered into the mass spectrometer through a nanoelectrospray ionization (nESI) source operating in positive ion mode (capillary voltage of 3 kV, cone voltage of 30 V, source temperature of 120ºC, nebulizing gas of 5.9 bar, nanoflow gas of 0.5 bar, and cone gas flow of 1 L/h). Mass spectrometry of peptide ions was performed in resolution data-independent acquisition mode (MS^E^). Mass-to-charge ratio (*m/z*) values of precursor ions (range of 100 and 2000 *m/z*) and product ions were obtained by ramping up collision energy (CE) from 0 to 20-45 V within 0.5 s, respectively. Prior to fragmentation processes, IMS was carried out to separate similar precursor ions with the following parameters: HE collision energy (in transfer CE ramp) of 19–45 V, IMS wave velocity ramping of 1000–500 m/s, and the default quad profile. For internal mass calibration, Glu-fibrinopeptide B (GFP; Waters) was sprayed into the mass spectrometer every 60 s.

For nLC-Q-orbitrap analysis, peptides eluted from the analytical column of the nLC were delivered into the mass spectrometer through a nESI source operating in positive ion mode (capillary voltage of 2.4 kV, source temperature of 250ºC). Full MS scans were acquired between 400 and 2000 *m/z* and ten most abundant ions were fragmented by higher energy collisional dissociation (HCD): the resolution for full MS of 70,000, the precursor ion isolation window of ±0.8 *m/z*, a normalized collision energy (NCE) of 27, the resolution for the product ion scan of 17,500, dynamic exclusion for 30 s, the discarded charge states of 1 and no less than 6, and automated gain control (AGC) at 1.0×10^6^ for both full MS scan and product ion scan.

Parameters of ProteinLynx Global Server Workflow Designer

ProteinLynx Global Server (PLGS, v3.0.2, Waters, Milford, MA) workflow designer parameters were set as follows: 3 minimum fragment ion matches per peptide, 7 minimum fragment ion matches per protein, 2 minimum peptide matches per protein, 1 missed cleavages, automatic mass tolerance for precursor ions and fragment ions, false discovery rate (FDR) threshold of 1%, and variable modifications of carbamidomethyl C and deamidated N.

Proteome Discoverer (v2.1, Thermo Scientific, Waltham, MA) parameters were set as follows: 2 minimum peptide length, 144 maximum peptide length, 1 missed cleavage, mass tolerance for precursor ions of 10 ppm, mass tolerance for product ions of 0.6 Da, false discovery rate (FDR) threshold of 1%, and variable modifications of carbamidomethyl C and deamidated N.
